# Neutrophils in the Pathogenesis of Rheumatic Diseases

**DOI:** 10.2478/rir-2022-0020

**Published:** 2022-10-20

**Authors:** Jia Tong Loh, Kong-Peng Lam

**Affiliations:** 1 Singapore Immunology Network, Agency for Science, Technology and Research, S138648 Singapore, Republic of Singapore; 2 Department of Microbiology and Immunology, Yong Loo Lin School of Medicine, National University of Singapore, S117545 Singapore, Republic of Singapore; 3 School of Biological Sciences, College of Science, Nanyang Technological University, S637551 Singapore, Republic of Singapore

**Keywords:** ANCA-associated vasculitis, neutrophils, neutrophil extracellular traps, rheumatoid arthritis, systemic lupus erythematosus

## Abstract

Rheumatic diseases, including rheumatoid arthritis (RA), systemic lupus erythematosus (SLE), and anti-neutrophil cytoplasmic antibody (ANCA)-associated vasculitis (AAV), are a group of auto-inflammatory disorders associated with substantial morbidity and mortality. One unifying feature of these diseases is the presence of abnormal neutrophils exhibiting dysregulated neutrophil extracellular trap (NET) release, reactive oxygen species (ROS) production, degranulation, and pro-inflammatory cytokines secretion. Moreover, the release of autoantigens associated with NETs promotes the generation of autoantibodies and a breakdown of self-tolerance, thereby perpetuating inflammation and tissue injury in these patients. In recent years, targeted therapies directed at neutrophilic effector functions have shown promising results in the management of rheumatic diseases. In this review, we will highlight the emerging roles of neutrophils in the onset and progression of rheumatic diseases, and further discuss current and future therapeutic approaches targeting the pathogenic functions of neutrophils, which can modulate inflammation and hence improve patients’ survival and quality of life.

## Introduction

Rheumatic diseases comprise a set of heterogeneous autoimmune disorders affecting predominantly the joints, tissues, and organs. Among them, the most common pathologies include rheumatoid arthritis (RA), systemic lupus erythematosus (SLE), and vasculitides.^[1]^ Over the past few decades, accumulating evidence points to the involvement of aberrant and abnormal neutrophils in the initiation and perpetuation of rheumatic diseases. In this review, we will provide an overview of the emerging roles of neutrophils in rheumatic diseases. We will discuss current knowledge on how neutrophils contribute to disease pathogenesis, and further highlight emerging neutrophil-based strategies for effective disease management.

## Neutrophil Development

Neutrophils are short-lived cells, with an estimated circulating half-life of <24 h. During steady state, they are continuously being replenished to maintain constant and sufficient numbers in the bloodstream for the containment of invading pathogens. Development of neutrophils begins from self-renewing hematopoietic stem cell (HSC) in the bone marrow, which differentiates into multipotent progenitor (MPP) and subsequently, granulocyte-monocyte progenitor (GMP). Within the GMP population, neutrophil-committed CD34^hi^CD106^−^CD11b^lo^ proNeu1 subset progressively differentiates into a transitional CD34^lo^CD106^+^CD11b^hi^ proNeu2 stage in transcriptional factor C/EBPɛ-dependent manner, before giving rise to the committed proliferative neutrophil precursor (preNeus). The preNeus (Ly6G^lo/+^CXCR2^−^c-kit^+^CXCR4^+^) then forms an intermediate non-proliferating immature neutrophil population (Ly6G^lo^CXCR2^−^CD101^−^) before terminally differentiating into functionally mature Ly6G^+^CXCR2^+^CD101^+^ neutrophils.^[2,3,4]^

While mature neutrophils are released into circulation to support their daily turnover, a large proportion of them are retained in the bone marrow as a reserve. Under inflammatory and diverse autoimmune conditions when large amounts of mature neutrophils are mobilized, signaling by the chemokine receptor CXCR2 facilitates the rapid egress of this neutrophil reserve within hours. However, during severe systemically disseminated infection or pathological conditions (for instance, myeloablation induced by chemotherapy) when neutrophils are consumed in large quantities, emergency granulopoiesis can be initiated as a compensatory mechanism to re-establish neutrophil homeostasis. This process is characterized by the selective expansion of proNeu1 and preNeus, as well as the release of immature and mature neutrophils from the bone marrow into circulation.^[5]^

### Effector Functions of Neutrophils

Neutrophils express a variety of pattern recognition receptors (PRRs) involved in the direct sensing of pathogen-and danger-associated molecular patterns (PAMPs and DAMPs), including Toll-like receptors (TLRs), C-type lectin receptors (CLRs), Nod-like receptors (NLRs), and RIG-like receptors (RLRs). These receptors enable them to respond almost instantly to pathogen invasion and diverse inflammatory stimuli in the tissue environment. Upon receptor activation, a series of complex and diverse signal transduction pathways will be triggered, which culminate in the mounting of antimicrobial immune responses, such as phagocytosis, reactive oxygen species (ROS) production, degranulation, and neutrophil extracellular trap (NET) release.^[6]^ Although these processes are pertinent for the elimination of invading microorganisms, prolonged and exaggerated activation of neutrophils could lead to hyperinflammation and tissue damage, indicating the importance of tight regulation to ensure a delicate balance between protective and pathological immune responses.

### Phagocytosis

Phagocytosis is one of the key microbicidal activities of neutrophils which helps eliminate microbes and foreign particles from the host. Phagocytosis can be initiated upon the interaction of invading microbes with non-opsonic phagocytic receptors such as Dectin-1 and Mincle expressed on the neutrophil cell surface. However, it is most efficient when pathogens are coated by host-derived opsonins such as immunoglobulin (Ig) G and complements, which facilitate their recognition by Fc receptors (FcR) and complement receptors (CRs), respectively. During FcR-mediated phagocytosis, neutrophils extend their ruffle-like pseudopods to envelope the IgG-opsonized target in a zippering process.^[7]^ On the other hand, rapid protrusion of a phagocytic cup is necessary for the engulfment of a complement-coated target.^[8,9]^ These led to the formation of a nascent phagosome, which matures upon fusion with preformed granules in the cytoplasm in a calcium-dependent manner.^[10]^ These granules are required to deliver a plethora of effector molecules, such as lytic enzymes, Nicotinamide adenine dinucleotide phosphate (NADPH) oxidase (NOX2), and antimicrobial peptides, to the phagosome to mediate pathogen killing and removal of apoptotic cells.

### Degranulation

Neutrophils can secrete an array of effector molecules encapsulated in granules into the extracellular environment and phagosomes to destroy invading pathogens. Four distinct granule subsets, namely the primary or azurophilic granules, the secondary or specific granules, the tertiary or gelatinase granules, and the secretory vesicles, are found in neutrophils. Azurophilic granules are packed with peptides and proteins which confer potent anti-microbial activity through oxidative as well as non-oxidative means, including myeloperoxidase (MPO), defensins, bactericidal/permeability-increasing protein (BPI), cathepsin G, elastase and serine proteases. Granule-derived MPO catalyzes the formation of hypochlorous acid (HOCl) and other cytotoxic oxidants, while BPI targets Gram-negative bacteria by binding to lipopolysaccharide (LPS) to neutralize their proinflammatory properties and promote phagocytosis. These granules primarily release their contents into the phagosome for the elimination of internalized pathogens. On the other hand, specific granules fuse predominantly with the plasma membrane to deliver their cargo extracellularly. These granules contain high levels of iron-binding protein lactoferrin, as well as anti-microbial peptides cathelicidin and LL-37. These peptides and proteins are usually stored in an inactive form in the granules and are activated by proteolytic cleavage upon secretion. Tertiary granules contain matrix metalloproteinase 9 (MMP9), beta-2 microglobulin, and various receptors and adhesion proteins to mediate the adhesion and penetration of neutrophils as they extravasate from the endothelium into the inflamed tissue. Secretory granules are endocytic vesicles containing membrane-associated receptors such as CR1/CR3, formylmethionyl-leucyl-phenylalanine (fMLP) receptors, and FcRs which play critical roles during early inflammation. During degranulation, receptor-mediated signaling triggers an elevated calcium signaling, which induces the granules to translocate to the phagosomal or plasma membrane through actin cytoskeleton remodeling and microtubule assembly. Following this, the granules will tether, dock, and fuse with the lipid bilayer membrane to release their contents into the phagosome or extracellular environment. Among the granules, secretory vesicles are most readily released from neutrophils, followed by tertiary granules, secondary granules, and finally azurophilic granules.^[11]^

### ROS Release

Neutrophils generate a strong oxidative burst in response to various stimuli such as phagocytosis and bacterial components for effective antimicrobial defense. Upon activation, NOX2 complex assembles itself on cellular membranes such as plasma membrane and membranes of the phagosomes and secretory vesicles to produce large amounts of superoxide. Upon release into the extracellular environment or phagolysosome following phagocytosis, the superoxide will be spontaneously or enzymatically dismutated to hydrogen peroxide, and MPO can further convert it into other secondary oxidants such as HOCl. Although ROS can induce direct oxidative killing, most of their bactericidal properties stem from their ability to augment pro-inflammatory cytokine production, degranulation, and NETosis.^[12]^

### NETosis

**Figure 1:**
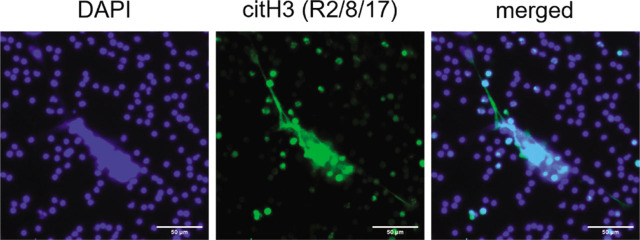
Release of NETs by human neutrophils in response to PMA. NETs are identified by DAPI (blue) and citrullinated histone 3 (R2/8/17) (green) staining. Scale bar, 50 μm. NETs, neutrophil extracellular traps. PMA, Phorbol-12-myristate-13-acetate.

NETs are large, extracellular, web-like structures composed of decondensed chromatin and granule proteins (including neutrophil elastase, MPO, calprotectin, and defensins) which are extruded from the neutrophils in response to large pathogens that cannot be phagocytosed.^[13]^ (Figure 1) However, small bacteria which evade phagocytosis through the formation of large aggregates or interfering with phagosome maturation can also induce NETs release. During NETosis, MPO activates neutrophil elastase in a ROS-dependent manner, and this promotes their release from the azurophilic granules into the cytoplasm to facilitate degradation of the actin cytoskeleton, thereby blocking phagocytosis. NE is then translocated to the nucleus, where it degrades histones and lamin to disrupt the chromatin packaging and nuclear envelope. In addition, protein-arginine deiminase type 4 (PAD4) is required for histone citrullination by converting amine groups on arginine to ketones, thereby leading to chromatin decondensation. The decondensed chromatin, together with the damaged nuclear lamina, contributes to the destruction of nuclear envelope and the subsequent release of chromatin into the cytoplasm.^[14]^ Activation of pore-forming protein gasdermin D protein (GSDMD) by caspase-11 has also been reported to induce nuclear delobulation, DNA expansion, and plasma membrane rupture to elicit NETosis.^[15]^ Although NETs release is a potent mechanism to combat invading pathogens, excessive production of NETs can lead to tissue damage and occlusion of vasculature. NETs can also serve as a source of autoantigens in various autoimmune rheumatic diseases, such as RA and SLE, which will be discussed in detail below.

### Role of Neutrophils in Rheumatic Diseases

While neutrophils play a cardinal role in anti-microbial defense, prolonged and excessive activation of neutrophils can lead to devastating consequences, such as cell lysis, tissue damage, and exacerbated inflammatory responses. In recent years, a growing body of evidence has implicated neutrophils in the onset and progression of various rheumatic diseases, including RA, SLE, and anti-neutrophil cytoplasmic antibody (ANCA)-associated vasculitis (AAV). In this section, we will outline current knowledge on the role of neutrophils in the pathogenesis of these rheumatic diseases.

### Rheumatoid Arthritis

Activated neutrophils are detected in the circulation of RA patients as compared to healthy individuals, and they can persist for several days and accumulate in large numbers within the RA synovial fluid and pannus, thus contributing to RA inflammation and joint destruction. In line with this, neutrophil depletion has been shown to inhibit the onset and ameliorate disease severity of experimental arthritis in mice, demonstrating their importance in the initiation and progression of RA.^[16,17]^ Recognition of immune complexes such as rheumatoid factor (RF) within the synovial fluid and on the articular cartilage surface by FcRs triggered the activation of neutrophils, leading to the production of pro-inflammatory cytokines such as B cell-activating factor (BAFF) and receptor activator of nuclear factor kappa B ligand (RANKL) which promote B cell activation and autoantibodies production, as well as the differentiation of osteoclast, respectively. Moreover, chemokine secretion by the activated neutrophils further enhances neutrophil infiltration into the RA joint, thereby amplifying the inflammatory immune response. In the RA joint, neutrophils have been reported to express major histocompatibility complex II (MHC II) to facilitate antigen presentation to and proliferation of T lymphocytes.^[18]^ The activated neutrophils also undergo degranulation in response to immune complexes to release contents like neutrophil elastase and collagenase which mediate cleavage of collagen, elastin and lubricin, resulting in cartilage damage.^[19]^ Abundant MPO has also been detected in the inflamed synovium of RA patients, leading to neutrophil recruitment, amplification of inflammation, and expansion of synovial fibroblasts.^[20]^ In addition, ROS production by neutrophils in RA patients can alter IgG to generate neo-epitopes which may induce circulating RA autoantibodies.^[21]^ Furthermore, neutrophils isolated from RA patients show enhanced NETs formation.^[22]^ Their sera contain high levels of anticitrullinated protein antibodies (ACPA) and RF which can induce NETosis, and this promotes the further generation of autoantigens in the form of citrullinated proteins (e.g, histones and vimentin present in NETs), hence perpetuating inflammation and tissue damage.^[22]^ Collectively, these mechanisms can fuel inflammatory arthritis and joint destruction, thereby driving disease pathogenesis and manifestations of RA.

### Systemic Lupus Erythematosus

While SLE is long known to be associated with dysregulated B and T cell responses, the pathogenic role of neutrophils in SLE has been increasingly recognized over the past decade. SLE patients have a characteristic increase in the numbers of immature, low-density neutrophils (LDNs) in their peripheral blood,^[23]^ and these SLE-derived LDNs adopt an activated phenotype with augmented production of type I interferon (IFN), Tumor necrosis factor alpha (TNFα) and IFNγ which contribute to disease pathogenesis. In addition, enhanced apoptosis, together with the impaired clearance of these neutrophils, led to the release of autoantigens and the generation of autoantibodies such as ANCAs in the patients, thereby promoting auto-inflammation. Neutrophils from SLE patients also tend to undergo spontaneous NETosis, resulting in the release of LL-37, citrullinated histones, neutrophil elastase, and MPO which are attached to the NET chromatin fibers.^[24,25]^ These further serve as autoantigens which can in turn activate plasmacytoid dendritic cells (pDCs) to produce large amounts of IFNα, thereby triggering a self-amplifying pathogenic loop.^[26]^ Moreover, NETs can mediate cardiovascular atherosclerotic complications in SLE patients by promoting endothelial damage through activation of the endothelial MMP-2. MPO and nitric oxide synthase present in NETs can further oxidize high-density lipoprotein to make it proatherogenic. In the vasculature, platelets can aggregate on and cooperate with NETs to enhance thrombosis. As such, the aberrant functions of neutrophils play an important part in promoting chronic inflammation and cardiovascular morbidity in SLE patients. Recently, neutrophil ferroptosis, an iron- and lipid-peroxidation-dependent programmed cell death, has been shown to drive neutropenia during SLE. In this case, autoantibodies and IFNα in the serum of SLE patients suppress glutathione peroxidase 4 (GPX4) expression through the calcium/calmodulin kinase IV (CaMKIV)/cAMP response element modulator (CREM)α signaling axis, thereby enhancing the production of lipid-ROS to induce ferroptosis, a major form of neutrophil cell death during SLE.^[26]^

### Anti-neutrophil Cytoplasmic Antibody-Associated Vasculitis

AAV refers to a group of diseases characterized by the inflammation of small blood vessels associated with necrotizing neutrophils. Pathogenesis of AAV is driven primarily by ANCAs targeting proteinase 3 (PR3) or MPO of neutrophils, and disease onset can be attributed to various genetic and environmental factors, including increased expression of PRTN3 gene or exposure to infectious pathogens such as *Staphylococcus aureus*. Upon priming by pro-inflammatory mediators like TNF, IL-1β and complement C5a, neutrophils express ANCA target antigens MPO and PR3 on their cell surfaces to facilitate ANCA binding. Concurrently, FcγRs on neutrophils engage the Fc portion of ANCAs to completely activate the neutrophils. Together, these series of events promote ROS production by and degranulation of neutrophils, which can directly damage vascular endothelial cells. ANCAs also activate neutrophils to undergo NETosis, releasing MPO and PR3 autoantigens which further amplify inflammation. Moreover, NETs can mediate endothelial injury and vascular inflammation in these patients by triggering the alternative complement pathway to produce complement factor 5a (C5a), a powerful chemoattractant for neutrophils. In addition, the presence of anti-NETs autoantibodies in AAV patients impairs the degradation and clearance of NETs, further contributing to the damage to small blood vessels. Collectively, these phenomena demonstrate the pathogenic role of neutrophils in AAV development and their associated long-term cardiovascular risk.^[27,28,29]^

### Neutrophil-Associated Therapies for Rheumatic Diseases

**Figure 2:**
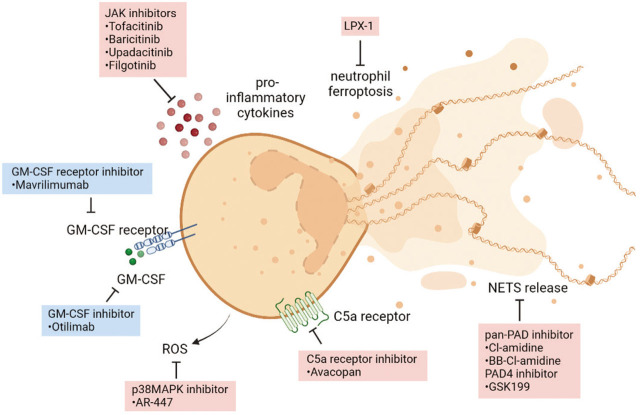
Overview of current and future therapies targeting neutrophil functions and survival. Biologics are shown in blue, and small molecule inhibitors are shown in red.

Despite our growing knowledge of the pathophysiology of rheumatic diseases as well as the availability of specific biologic therapies, broad-spectrum anti-inflammatory glucocorticoids continue to be the first-line treatment for rheumatic patients. Even though glucocorticoids play a pivotal role in the management of inflammatory autoimmune diseases, the adverse side effects associated with their prolonged usage are common and well-established.^[30]^ Since accumulating evidence suggests that neutrophils are the major orchestrator of inflammation and tissue damage in rheumatic diseases, it may be an attractive strategy to target neutrophils and their effector functions for disease management (Figure 2 and Table 1).

**Table 1:** Existing and potential drugs targeting neutrophils in rheumatic diseases

**Neutrophil mechanism**	**Molecular target**	**Agent**	**Disease**	**Current stage of development**	**Ref**
NETosis	PAD	Cl amidine BB-Cl amidine	AAV, SLE, RA	Preclinical	[31–32]
	PAD4	GSK199	RA	Preclinical	[32,33]
ROS production	p38MAPK	AR-447	AAV	Preclinical	[34]
Pro-inflammatory cytokines production	JAK	Tofacitinib Baricitinib Upadacitinib	RA	FDA approved	[35,36]
		Filgotinib	RA	Phase III	[37,38]
Survival and migration	GM-CSF	Otilimab	RA	Phase II	[39]
	GM-CSF receptor	Mavrilimumab	RA	Phase II	[40,41]
Activation and recruitment	C5a receptor	Avacopan	AAV	FDA approved	[42]
Ferroptosis	unknown	LPX-1	SLE	Preclinical	[26]

AAV, anti-associated vasculitis; GM-CSF, Granulocyte-macrophage colony-stimulating factor; LPX-1, liproxstatin-1; PAD4, protein-arginine deiminase type 4; RA, rheumatoid arthritis; ROS, reactive oxygen species; SLE, systemic lupus erythematosus.

### Inhibition of NETosis

NETs play an important pathogenic role in the initiation and progression of several rheumatic diseases as outlined above. Hence, potential candidates that can block NETosis are currently being explored in various preclinical studies. PAD4 is an enzyme crucial for NET formation through catalyzing histone citrullination. Indeed, PAD4 inhibition using pan-PAD inhibitors like Cl-amidine or PAD4-specific inhibitors such as GSK199 can disrupt NETosis and hence ameliorate disease severity in mouse models of SLE, RA, and AAV.^[31,32,33]^ Moreover, recent studies revealed that PAD4-specific inhibitors have the advantage of few off-target effects, further supporting their utility as treatment options for rheumatic patients.^[43]^ However, the efficacy of PAD4 inhibitors in the treatment of rheumatic diseases remains to be determined in clinical trials.

### Inhibition of ROS Production

Since oxidative burst by neutrophils can trigger tissue injury and damage in rheumatic diseases, management of oxidative stress has been explored as a potential therapy. Inhibition of p38 mitogen-activated protein kinase (p38MAPK) was previously shown to suppress neutrophil respiratory burst and hence prevent neutrophil activation by ANCAs in a mouse model of AAV.^[34]^

### Inhibition of Pro-inflammatory Cytokines Production

Given that neutrophil activation leads to pro-inflammatory cytokines secretion, targeting these mediators represents a way to treat rheumatic diseases. Although biologics targeting pro-inflammatory cytokines (eg. Anti-TNFα) are highly efficacious in the treatment of rheumatic diseases, their development has been greatly hampered by their cost of production, route of administration, as well as safety profile due to the immunogenic nature of these biologics.^[35]^ As such, targeting signaling pathways that function downstream of cytokine receptors has been explored as alternative immunotherapeutic strategies. The JAK-STAT pathway plays a major role in transducing signals from a myriad of cytokines. Currently, three clinically approved small molecule JAK inhibitors, Tofacitinib, Baricitinib, and Upadacitinib, are in the market for RA treatment.^[34,35]^ In addition, next-generation JAK inhibitor Filgotinib is being evaluated in several phase III clinical trials for RA, and the results have been encouraging.^[37,40,44]^

### Inhibition of Neutrophil Survival

Granulocyte-macrophage colony-stimulating factor (GM-CSF) is an important growth factor that promotes neutrophil survival. Targeting the effects of GM-CSF with otilimab or mavrilimumab has shown promising results in clinical trials for RA.^[38,39]^

### Inhibition of Neutrophil Activation and Recruitment

C5a is a product of the alternative complement pathway which mediates neutrophil recruitment and activation upon receptor binding. Avacopan, a small molecule inhibitor of the C5a receptor, has recently been approved by U.S. Food and Drug Administration (FDA) for the treatment of AAV.^[42]^

### Inhibition of Neutrophil Ferroptosis

Neutrophil ferroptosis plays a critical role in the pathogenesis of SLE. Indeed, inhibition of ferroptosis by liproxstatin-1 (LPX-1) has been shown to rescue neutrophil cell death and alleviate disease severity in a mouse model of SLE.^[26]^

### Outlook

Advancement in the field of rheumatology has led to considerable progress in disease management over the past few decades. With appropriate treatment, clinical remission has become a realistic therapeutic goal for most rheumatic patients. However, the current treatment strategy relies mainly on the usage of glucocorticoids to suppress inflammation, and long-term immunosuppression is commonly associated with serious adverse side effects like infection and cancer. As such, a better understanding of the molecular pathways underlying each disease's onset and progression is necessary for the development of next-generation targeted therapies. In this review, we have discussed the role of neutrophils in driving rheumatic diseases and highlighted several promising pathways and signaling molecules that can be targeted to suppress their deleterious functions. However, this is a challenging endeavor since neutrophils are the first line of defense against infection and hence play a fundamental role in host immune responses. Ideally, their effector functions need to be targeted specifically under pathological conditions. Recently, our laboratory has identified Dok3 to be a key negative regulator of neutrophilic effector mechanisms, and it functions in a context-dependent manner downstream of different immunoreceptors via interaction with a distinct set of signal transducing molecules.^[45,46,47]^ Thus, it is tempting to speculate that Dok3 may regulate neutrophil responses during rheumatic diseases as well. A deeper understanding of the Dok3 signaling pathway could potentially reveal binding partners which can be targeted for future treatment of rheumatic diseases. In addition, less toxic immunomodulatory approaches may be explored as therapeutic options for rheumatic diseases, as opposed to the use of broad-spectrum non-specific immunosuppressive agents. For instance, mesenchymal stem cell (MSC) exosomes, which possess unique immunomodulatory properties, have emerged as superior, well-tolerated candidates for therapeutics in recent years. Several preclinical studies have demonstrated their potential in suppressing joint inflammation during RA,^[48]^and we have previously reported their ability to suppress NETosis in neutrophils in response to complement activation.^[49]^ However, future studies are warranted to understand the mechanistic effect of MSC exosomes on neutrophils during rheumatic diseases for them to be approved as potential anti-rheumatic therapeutics.

While achieving remission represents an important milestone in the management of rheumatic diseases, the ultimate goal is to develop a cure since relapse is likely to occur once an anti-rheumatic treatment is withdrawn.^[50]^ To tackle this goal, we will need to shift our treatment strategies from the targeting of neutrophil effector functions that elicit the immediate manifestations, to the identification of disease drivers which trigger the long-standing inflammation. Moving forward, studies will need to decipher the pathophysiology of rheumatic diseases which activates downstream pro-inflammatory effector pathways. The neutrophil is likely to be a key immunological driver of rheumatic diseases, and we propose future work to focus on investigating the molecular signatures of neutrophils associated with remission and cure. Single-cell RNA-seq of these cells in healthy and pathological conditions may yield new targets and pathways for disease intervention. These studies are likely to reshape the landscape of rheumatic disease management over the next few years, thus improving the quality of life for patients.
